# The impact of oral diseases in cirrhosis on complications and mortality

**DOI:** 10.1002/jgh3.12489

**Published:** 2021-01-12

**Authors:** Lea Ladegaard Grønkjær, Palle Holmstrup, Peter Jepsen, Hendrik Vilstrup

**Affiliations:** ^1^ Department of Hepatology and Gastroenterology Aarhus University Hospital Aarhus Denmark; ^2^ Department of Gastroenterology Hospital of South West Jutland Aarhus Denmark; ^3^ Section of Periodontology, Department of Odontology Faculty of Health and Medical Sciences, University of Copenhagen Copenhagen Denmark; ^4^ Department of Clinical Epidemiology Aarhus University Hospital Aarhus Denmark

**Keywords:** cirrhosis, disease course, mortality, oral diseases, oral health

## Abstract

**Background and Aim:**

The aims of this study were to describe the prevalence of various oral diseases and to examine the association of the oral diseases with complications and mortality of cirrhosis.

**Methods:**

A total of 184 cirrhosis patients were enrolled and were followed up for 2 years. They underwent oral clinical and radiographic examination. At study entry, the associations between oral diseases with nutrition, inflammation, and cirrhosis complication status were examined. Then, the associations of oral diseases with all‐cause and cirrhosis‐related mortality were examined using Cox regression to adjust for confounding by age, gender, smoking, alcohol use, alcoholic cirrhosis, cirrhosis complications, comorbidity, Child‐Pugh, and Model of End‐Stage Liver Disease (MELD) score.

**Results:**

At entry, 26% of the patients had gross caries, 46% periapical lesions, 27% oral mucosal lesions, and 68% periodontitis. Having one or more oral diseases was associated with a higher prevalence of cirrhosis complications (46.7 *vs* 20.5%), higher C‐reactive protein (28.5 mg/L *vs* 10.4 mg/L), and higher nutritional risk score (4 *vs* 3). Two‐thirds of the patients died during follow‐up. The patients with more than one oral disease had an increasingly higher all‐cause mortality (two diseases: hazard ratio [HR] 1.55, 95% confidence interval [CI] 1.02–1.98; three and four diseases: HR 1.75, 95% CI 1.05–3.24) and even higher cirrhosis‐related mortality (two diseases: HR 1.60, 95% CI 1.01–2.40; three and four diseases: HR 2.04, 95% CI 1.05–8.83) compared to those with no oral disease.

**Conclusion:**

In cirrhosis, having more than one oral disease was associated with more complications and with higher mortality.

## Graphical abstract

Poor oral health and oral diseases are prevalent in patients with cirrhosis. There is increasing interest in the connection between oral and general health. However, an understanding of the clinical significance of oral health in this patient group is lacking. Here, we show that patients with oral diseases had more cirrhosis complications than patients without oral diseases. Moreover, having oral disease was associated with higher mortality.

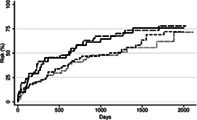

## Introduction

Over the past decades, evidence has accumulated to show that oral health is fundamental to general health.^1^ Thus, several studies describe an association between oral diseases and cancer, cardiovascular diseases, chronic kidney disease, diabetes mellitus, and rheumatoid arthritis. This body of evidence suggests that oral diseases due to bacterial contamination of the bloodstream and systemic low‐grade inflammation may negatively affect the course of systemic diseases, leading to higher morbidity and mortality.^2^, ^3^, ^4^, ^5^

Likewise, several studies show that patients with cirrhosis have poor oral health with a high prevalence of oral diseases compared to the general population,^6^, ^7^, ^8^, ^9^, ^10^ but the clinical significance in this patient group is still sparsely explored.

In previous studies, we focused on the association of one single oral disease, such as periapical lesions and periodontitis, with the complications and mortality of cirrhosis.^11^, ^12^, ^13^ However, often, patients have more than one oral disease, and the impact of multiple oral diseases on the clinical course of cirrhosis may be greater than that of only one such disease or even exceed the sum of the effects of individual diseases.^14^ Thus, an additively negative effect of comorbidity or multimorbidity has been shown on systemic diseases.^15^ Therefore, the aims of this study were to describe the prevalence of multiple oral diseases and their associations with cirrhosis complications, inflammation, and nutrition status at study entry in a cohort of cirrhosis patients and to examine the associations between oral diseases singly and in combination and all‐cause and cirrhosis‐related mortality.

## Methods

The patients were consecutively enrolled from March 2013 to November 2015 at the Department of Hepatology and Gastroenterology, Aarhus University Hospital, Denmark.^13^ All adult men and women with an established diagnosis of cirrhosis based on either liver biopsy and/or on clinical, biochemical, and ultrasonographic findings were included, regardless of whether their admission to the department was acute or scheduled and regardless of cirrhosis etiology and severity. We excluded those who could not speak or understand Danish, had dementia or were cognitively impaired including overt hepatic encephalopathy, had hepatocellular carcinoma or other malignant diseases, or had acute critical conditions. The patient cohort is described in our previous study,^13^ and the present study focuses on the effects of one or more oral diseases on the cohort's clinical course by including long‐term follow‐up data.

A total of 273 patients were assessed, of whom 89 were excluded due to the exclusion criteria (*n* = 49), lack of consent (*n* = 37), or death before the oral and radiographic examinations (*n* = 3).^13^

### *Clinical data*

Demographics, clinical information, and laboratory results at the time of inclusion were asked or collected from the patients' medical charts, including age, gender, marital status, occupational status, smoker status (current, former, never), present use of alcohol (yes or no) and alcoholic hepatitis, cirrhosis etiology, cirrhosis complication status (episodes of ascites and spontaneous bacterial peritonitis, hepatic encephalopathy, and/or variceal bleeding), and comorbidity. The comorbidities were classified, and a total score was calculated using the Charlson comorbidity index.^16^ Patients' nutritional risk was assessed using the standardized screening tool NRS‐2002.^17^ To gauge the severity of the cirrhosis disease, the Child‐Pugh score and the Model of End‐Stage Liver Disease (MELD) score was calculated.^18^ The inflammatory marker C‐reactive protein (CRP) was obtained from blood samples taken on the day of the oral and radiographic examination, and the number of patients receiving antibiotic treatment was calculated.

All patients were prospectively followed up in the department until death or study termination on 25 November 2018. No patient was lost to follow‐up. Dates of death were obtained from the Civil Registration System, an administrative registry continuously updated with vital status and dates of death for all Danish citizens.^19^ The cause of death up to and including 2018 was obtained from the Danish Register of Causes of Death, which contains information on all death certificates since 1943.^20^

At study entry, 12 (7%) of the patients were under evaluation for liver transplantation, and 2 (1%) underwent transplantation during the follow‐up.

### *Oral and radiographic examination*

At study entry, clinical and radiographic oral examinations were performed. This included registrations of present teeth and gross caries, that is, with advanced dental decay. Plaque was measured according to the criteria of Silness and Löe.^21^ Furthermore, a periodontal examination including full‐mouth registration was completed. The assessment of pocket depth, bleeding on probing, and gingival margin levels were registered at six sites on each tooth. Gingival recession was registered as a negative value. Clinical attachment level was calculated as the sum of the pocket depths and gingival level measurement. Pocket depths and clinical attachment level were rounded to the nearest mm. Periodontitis was defined using the definition developed by the working group of the Centre for Disease Control and Prevention in collaboration with the American Academy of Periodontology.^22^ Patients with severe or moderate periodontitis were grouped as having periodontitis. Moreover, the examination included an inspection of the oral cavity. Patients' oral mucosa was examined, and any oral mucosal lesion was described.

Three authorized dental hygienists were responsible for the oral examinations, and they were trained by a dentist specialized in periodontitis from the Department of Odontology, Aarhus University. Prior to and halfway through the study, the dental hygienists were calibrated for periodontal registrations. On both occasions, their inter‐ and intraexaminer reproducibility was assessed by calculation of Lin's concordance correlation coefficient and the degree of agreement according to the categories suggested by McBride.^23^, ^24^ We found moderate to substantial agreement, which was regarded as appropriate for the study purpose.

Periapical status was assessed by digital panoramic radiography, which was taken at the Department of Oral and Maxillofacial Surgery, Aarhus University Hospital, using a Planmeca ProMax 3D. The images were examined in a room with adjustable light using a computer with Planmeca Romexis software. Periapical lesions were defined as the presence of radiolucency in connection with the apical part of the root or widening of the apical part of the periodontal ligament space to greater than twice the normal width.^25^ A senior resident in oral and maxillofacial surgery examined and described the radiographies after training at the Department of Odontology, Aarhus University.

### *Ethical considerations*

The study was conducted in accordance with the Helsinki Declaration and approved by the Ethical Committee of Central Denmark Region (journal No. 1‐10‐72‐128‐12). Written informed consent was obtained from all participants. The participants were informed about their oral health status. In case of oral diseases or problems, the patient was recommended to contact a dentist for appropriate treatment.

### *Data analysis*

A categorical variable was calculated to combine the presence of four oral diseases (i.e. gross caries, oral mucosal lesions, periapical lesions, and periodontitis). This variable was classified as follows: no oral disease and one, two, three, and four oral diseases. Having three and four oral diseases was merged as only five patients had all four oral diseases, leaving four categories. The chi‐square test was performed to examine the association between no or one or more oral diseases and patients' gender, smoker status, alcohol use, cirrhosis etiology, and present cirrhosis complications. Due to nonnormally distributed data, the Kruskal‐Wallis test was performed to examine the association between no or one or more oral diseases and patients' age, comorbidities, cirrhosis severity, CRP, and nutritional risk score. To examine the association of oral diseases with mortality, a categorical all‐cause mortality variable, grouping alive and dead patients, was calculated. In addition, a cirrhosis‐related mortality variable was calculated. Cirrhosis‐related deaths were grouped as those patients with cirrhosis (K70.3, K74.6) or liver failure (K.70.4, K72) listed as the cause of death or as part of the events leading to death. All other deaths were classified as not cirrhosis related. Then, two sets of analyses were performed. First, a Cox proportional hazard regression analysis was used to estimate the association between the presence or absence of each one of the four oral diseases and all‐cause and cirrhosis‐related mortality. Second, Cox proportional hazards regression analysis was used to explore the association between the categorical oral disease variable and all‐cause and cirrhosis‐related mortality. The analysis was adjusted for confounding by age, male gender, smoker status, alcohol use, cirrhosis complications, comorbidity, and Child‐Pugh and MELD scores. Age, Charlson comorbidity index, Child‐Pugh, and MELD score were included as continuous, linear variables. The proportional hazards assumption was evaluated graphically using log–log plots of the survival curves. We used the cumulative incidence function to compute the cumulative all‐cause and cirrhosis‐related mortality, taking into account that they were competing risks.^26^ The data were analyzed using Stata version 12.0 (Stata Corp LP, College Station, TX, USA).

## Results

The median age of the 184 patients was 62 years (range 21–87), and two‐thirds were men (66%). The total follow‐up time was 427 years, with a median of 2 years per patient. During follow‐up, 123 patients (67%) died, whereas 101 (82%) died from cirrhosis‐related causes, including 3 from hepatocellular carcinoma. Twenty‐two patients (18%) died from other causes, with the most common being cardiovascular, infection or nonhepatic malignancy. Patients' clinical and demographic characteristics are summarized in Table 1.

**Table 1:** Clinical and demographical characteristics of the cirrhosis patients (*n* = 184)

**Age, year**, median (range)	62 (21–87)
**Gender**, *n* (%)	
Male	121 (66)
Female	63 (34)
**Marital status**, *n* (%)	
Single/divorced/widower	99 (54)
Married/cohabiting	85 (46)
**Occupational status**, *n* (%)	
Employed	13 (7)
Unemployed	31 (17)
Disability pensioner	50 (27)
Retired	90 (49)
**Smoker status**, *n* (%)	
Current	29 (16)
Former	83 (45)
Never	72 (39)
**Alcohol use**, *n* (%)	
Present alcohol use	70 (38)
No present alcohol use	114 (62)
Patients with alcoholic hepatitis at study entry (Glasgow alcoholic hepatitis score > 9)	3 (2)
**Cirrhosis etiology**, *n* (%)	
Alcohol	134 (73)
Autoimmune or cholestatic	20 (11)
Cryptogenic	22 (12)
Viral hepatitis B and/or C	8 (4)
**Cirrhosis complications**, *n* (%)	
Patients with present cirrhosis‐related complications	66 (36)
Ascites	44 (24)
Patients with spontaneous bacterial peritonitis at study entry	9 (5)
Hepatic encephalopathy	8 (4)
Variceal bleeding	14 (8)
**Comorbidity**, *n* (%)	
Charlson comorbidity index	7 (4)
3+	24 (13)
2	44 (24)
1	109 (59)
0	
Most frequent comorbidities	
Diabetes	35 (19)
Cardiovascular disease	33 (18)
Chronic lung disease	11 (6)
Renal disease	6 (3)
**Cirrhosis severity**, median (range)	
Child‐Pugh score	8 (5–12)
MELD score	11 (6–37)
**Inflammation status**	
C‐Reactive Protein Median (range)	13.3 mg/L (0.9–101.0 mg/L)
Patients receiving antibiotic treatment at study entry *n* (%)	22 (12)
**Nutritional status**, median (range)	
Nutritional Risk Score	3 (1–7)
**Mortality**, *n* (%)	
Deaths during follow‐up	123 (67)
Causes of death	
Cirrhosis‐related	101 (82)
Cardiovascular	8 (7)
Infection	4 (3)
Nonhepatic malignancy	3 (2)
Lung disease	2 (2)
Kidney insufficient	2 (2)
Unknown	2 (2)
Pancreatic insufficiency	1 (0.8)

Data are presented as number and percentage or median and range.

MELD, Model of End‐Stage Liver Disease.

### *Oral diseases*

Table 2 shows the oral health characteristics of the patients. The median number of teeth was 22, and 18% of the patients were edentulous. At entry, 26% had one or more teeth with gross caries (range 1–17), 46% had one or more teeth with periapical lesions (range 1–9), and 27% had one or more oral mucosal lesions. Most common of these were oral candidiasis (56%) and oral mucosal ulcers (24%). Furthermore, 68% had periodontitis. Of the patients, 24% had no oral disease, while 33% had one oral disease, 25% had two, 15% had three, and 3% had four oral diseases.

**Table 2:** Oral health characteristics of the cirrhosis patients

**Dental status**	
Number of teeth, median (IQR)	22 (6–27)
Edentulous patients (%)	18
Prevalence of gross caries in all examined teeth (%)	6
Patients with gross caries (%)	26
**Periodontal status**	
Sites with plaque (%)	89
Probing depth, mm, median (IQR)	3.02 (2.28–3.56)
Clinical attachment level, mm, median (IQR)	3.47 (2.60–4.04)
Sites with bleeding on probing (%)	39
Patients with severe periodontitis (%)	45
Patients with moderate periodontitis (%)	39
Patients with no‐or‐mild periodontitis (%)	16
**Oral mucosal status (%)**	
Patients with oral mucosal lesions (all diagnoses)	27
Candidiasis	56
Oral mucosal ulcers	24
Fissured or geographic tongue	20
**Periapical status**	
Prevalence of periapical lesions in all examined teeth (%)	4
Patients with periapical lesions (%)	46

Data are presented as percentage or median and interquartile range.

IQR, interquartile range.

There were no differences in age, gender, smoker status, alcohol use, comorbidity, and cirrhosis severity in patients with one or more oral diseases compared to patients with no oral disease. However, there was a tendency that more patients with alcoholic cirrhosis had one or more oral diseases compared with nonalcoholic cirrhosis (81 *vs* 61%, *P* = 0.07).

### *Cirrhosis complications*

Sixty‐six patients (36%) had cirrhosis complications such as ascites (24%), hepatic encephalopathy (4%), and/or variceal bleeding (8%). Patients' median nutritional risk score was 3, and their median CRP was 13.3 mg/L. Having one and up to four oral diseases compared with no oral disease was associated with the presence of more cirrhosis complications (46.7 *vs* 20.5%), higher CRP (28.5 *vs* 10.4 mg/L), and nutritional risk score (4 *vs* 3) (Table 3).

**Table 3:** Association of oral diseases with cirrhosis complications

	Ascites, hepatic encephalopathy, and/or variceal bleeding (%)	C‐Reactive protein (IQR)	Nutritional risk score (IQR)
No oral disease	20.5%	10.4.1 mg/L (3.4 mg/L‐61.5 mg/L)	3 (2–5)
One oral disease	32.6%	12.1 mg/L (5.8 mg/L‐58.5 mg/L)	3 (2–5)
Two oral diseases	42.0%	14.5 mg/L (6.7 mg/L‐76.3 mg/L)	4 (2–6)
Three and four oral diseases	46.7%	28.5 mg/L (8.0 mg/L‐132.5 mg/L)	4 (3–7)
*P*‐value	0.04	0.03	0.07

Data are presented as percentage or median and interquartile range.

IQR, interquartile range.

### *Mortality*

The presence of gross caries, oral mucosal lesions, periapical lesions, and periodontitis compared to no oral disease was associated with higher all‐cause mortality (gross caries: adjusted hazard ratio (HR) 1.10, 95% confidence intervals (CI) 0.99–1.05; oral mucosal lesions: adjusted HR 1.27, 95% CI 1.01–1.91; periapical lesions: adjusted HR 1.54, 95% CI 0.99–1.79; periodontitis: adjusted HR 2.23, 95% CI 1.10–4.92) and cirrhosis‐related mortality (gross caries: adjusted HR 1.26, 95% CI 0.75–2.09; oral mucosal lesions: adjusted HR 1.60, 95% CI 1.03–2.51; periapical lesions: adjusted HR 1.64, 95% CI 0.87–3.08; periodontitis: adjusted HR 3.19, 95% CI 1.14–8.90) (Table 4).

**Table 4:** Oral disease and risk of mortality

	All‐cause mortality		Cirrhosis‐related mortality	
	Crude HR (95% CI)	Adjusted HR (95% CI)	Crude HR (95% CI)	Adjusted HR (95% CI)
Gross caries *versus* no gross caries (*n* = 151)	1.06 (0.67–1.69)	1.10 (0.99–1.05)	1.21 (0.74–1.97)	1.26 (0.75–2.09)
Oral mucosal lesions *versus* no oral mucosal lesions (*n* = 184)	1.72 (1.16–2.54)*	1.27 (1.01–1.91)*	1.94 (1.28–2.95)*	1.60 (1.03–2.51)*
Periapical lesions *versus* no periapical lesions (*n* = 110)	1.85 (1.09–3.13)*	1.54 (0.99–1.79)	1.99 (1.10–3.57)*	1.64 (0.87–3.08)
Periodontitis *versus* no periodontitis (*n* = 151)	3.22 (1.49–6.97)*	2.23 (1.10–4.92)*	4.16 (1.68–12.63)*	3.19 (1.14–8.90)*

* *P* < 0.05.

Associations are expressed as HR with CI and presented without adjustment and with adjustment for age, gender, smoker status, alcohol use, alcoholic cirrhosis, comorbidity, and Child‐Pugh and MELD scores.

CI, confidence interval; HR, hazards ratios; MELD, Model of End‐Stage Liver Disease.

**Figure 1:**
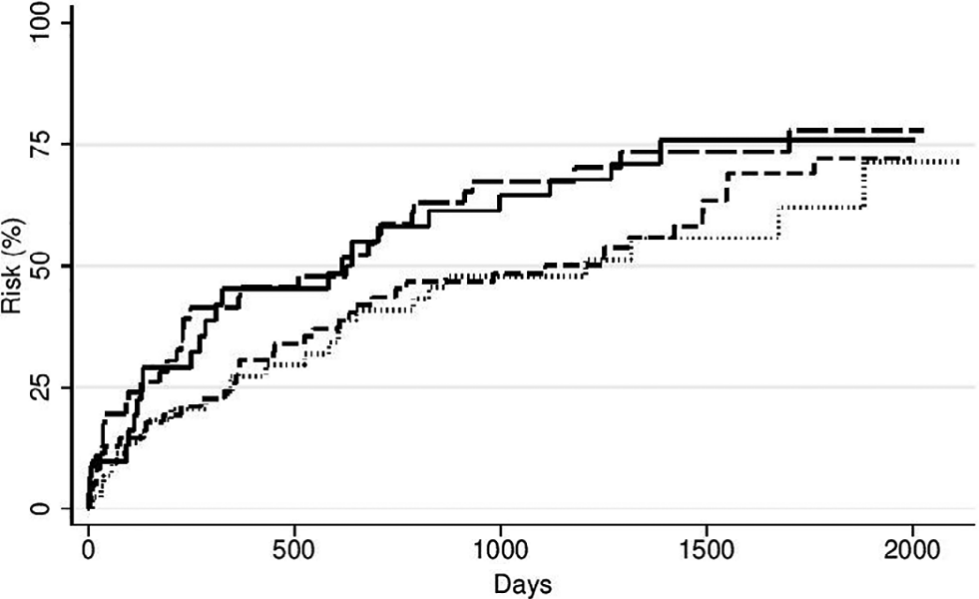
Cirrhosis‐related mortality caused by oral diseases. (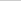), No oral disease; (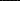), two oral diseases; (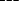), one oral disease; (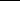), three and four oral diseases.

When combining the oral diseases into one variable, patients with any single one of the four oral diseases had the same all‐cause and cirrhosis‐related mortality as the patients with no oral disease (all‐cause: adjusted HR 1.05, 95% CI 0.33–1.76; cirrhosis‐related: adjusted HR 1.01, 95% CI 0.55–2.40) (Table 5). The patients with more than one oral disease had an increasingly higher all‐cause mortality (two diseases: adjusted HR 1.55, 95% CI 1.02–1.98; three and four diseases: adjusted HR 1.75, 95% CI 1.05–3.24) and an even higher cirrhosis‐related mortality (two diseases: adjusted HR 1.60, 95% CI 1.01–2.40; three and four diseases: adjusted HR 2.04, 95% CI 1.05–8.83) compared to the patients with no oral disease (Table 5 and Fig. 1).

**Table 5:** Multiple oral disease and risk of all‐cause and cirrhosis‐related mortality

	All‐cause mortality		Cirrhosis‐related mortality	
	Crude HR (95% CI)	Adjusted HR (95% CI)	Crude HR (95% CI)	Adjusted HR (95% CI)
One oral disease *versus* no oral disease	1.10 (0.67–1.20)	1.05 (0.63–1.76)	1.08 (0.60–1.93)	1.01 (0.55–2.40)
Two oral diseases *versus* no oral disease	1.51 (1.01–2.67)*	1.55 (1.02–1.98)*	1.81 (1.06–3.37)*	1.60 (1.01–2.40)*
Three and four oral diseases *versus* no oral disease	1.59 (1.16–2.67)*	1.75 (1.05–3.24)*	1.89 (1.31–3.35)*	2.04 (1.05–8.83)*

* *P* < 0.05.

Associations are expressed as HR with CI and presented without adjustment and with adjustment for age, gender, smoker status, alcohol use, alcoholic cirrhosis, comorbidity, and Child‐Pugh and MELD scores.

CI, confidence interval; HR, hazards ratios; MELD, Model of End‐Stage Liver Disease.

## Discussion

This cohort study showed that cirrhosis patients had highly prevalent multiple oral diseases, which were associated with increases in cirrhosis complications, CRP, and nutritional risk score. In addition, the presence of more than one oral disease was associated with higher all‐cause and cirrhosis‐related mortality, increasing with the number of diseases.

The most important strengths of our study are its prospective design, the systematic and detailed oral and radiographic examination, and the complete follow‐up. It is also a strength that we studied a broad spectrum of cirrhosis patients with regard to etiology and severity. The distributions of age, gender, and cirrhosis etiology correspond to recent Danish nationwide studies.^27^, ^28^ In addition, only a few patients had alcoholic hepatitis and spontaneous bacterial peritonitis and were receiving antibiotic treatment at study entry, which may have affected their short‐term mortality. A large fraction of the patients were classified as having cryptogenic cirrhosis, which may reflect non‐alcoholic steatohepatitis. However, this does not affect the results of this study. In addition, although we recruited from both local and referred patients, it remains a limitation that it is a single‐center study. Another limitation is the study design, which implies that it cannot be concluded that more oral diseases were in fact the cause of more complications and higher mortality or whether the oral diseases resulted from more advanced disease course. However, the lack of association at study entry between the Child‐Pugh and MELD scores and the presence of oral diseases favor the first assumption. We controlled for obvious confounding by statistical adjustments. We did not control for socioeconomic status, and although the Danish population is more homogenous with respect to income and disability status than most other countries,^27^, ^29^ we may have missed an aggravating effect of severe social deprivation.

Our study has no healthy control cohort, and our findings rely on internal analyses, with patients without oral disease serving as sick controls. The prevalence of oral diseases in our cohort was in line with other studies of cirrhosis patients^6^, ^7^, ^8^, ^9^, ^10^ but was very high compared to descriptions of the general population.^30^, ^31^ However, this comparison is uncertain because of variations in the reported background population in sampling procedures and the diagnostic criteria for oral diseases.

There was a tendency that patients with alcoholic cirrhosis had more oral diseases than those with non‐alcoholic cirrhosis. This is consistent with findings from other studies^9^, ^10^, ^32^ that consider non‐alcoholic cirrhosis patients to be more health conscious than those with alcoholic cirrhosis. However, autoimmune and cholestatic liver diseases are related to Sjögren's syndrome, which may cause oral dryness and favors oral diseases.^11^

The presence of multiple oral diseases was associated with higher CRP, indicating a degree of systemic inflammation activation beyond that associated with cirrhosis itself. A possible mechanism behind this includes bacterial translocation.^2^, ^33^, ^34^ This may conceivably act as a pathogenic link in the development of more cirrhosis complications and higher mortality, which explains our current and former findings.^12^, ^13^

In addition, the subgingival microbiota in cirrhosis patients seems to be markedly changed to include bacteria not normally associated with oral diseases. The acquired immune defect of cirrhosis seems to allow commensal bacteria to prevail and become pathogenic. Likewise, the immune defect may predispose patients to oral candidiasis.^35^

The presence of multiple oral diseases tended to be associated with an increased nutritional risk score. It is plausible that such diseases negatively impact the patients' food intake, contributing to the risk of malnutrition, a major and partly unexplained complication of cirrhosis, leading to higher morbidity and mortality.^32^, ^36^

The follow‐up part of our study demonstrates that periodontitis was associated with a higher risk of mortality compared to the other oral diseases. In studies of patients with cardiovascular diseases and diabetes mellitus, it has been suggested that periodontitis may lead to more complications and higher mortality due to spreading of oral bacteria and bacterial products, either by aspiration or through the bloodstream. Likewise, spillover of inflammatory cytokines may induce and perpetuate systemic effects.^2^, ^3^, ^9^ It may very well be the same for patient with cirrhosis.

The follow‐up part also showed that the presence of multiple oral diseases was linked to higher all‐cause mortality, largely driven by higher cirrhosis‐related mortality, an effect increasing with the number of oral diseases. This is in accordance with other studies suggesting oral disease as a ‘nontraditional’ prognostic factor influencing the clinical course of liver disease.^37^, ^38^ The one‐disease threshold and dose‐dependent effect of multiple oral diseases we describe have not previously been investigated in patients with cirrhosis but are consistent with studies of patients with chronic kidney disease.^39^

The mechanisms behind the linking of oral disease to the worsening of cirrhosis needs further investigations, but the prevalence part of our study may direct attention toward inflammation activation and malnutrition, leading to worse disease course. Anyway, our findings show that more clinical attention should be directed toward oral health status and care. We have shown that cirrhosis patients live with oral hygiene neglect,^32^ but currently, only patients awaiting liver transplantation are undergoing an oral and radiographic examination. There is a need to evaluate the effect of improved oral care in intervention studies. Promising results are described in a small pilot study of hepatitis C patients,^40^ and a recent study indicates an improvement in oral and gut microbiota dysbiosis, cirrhosis severity, and systemic inflammation activation after improved oral care and treatment by simple means.^41^ It is worth noting that two‐thirds of our patients died during the relatively short observation time, so there is an urgent need for all sorts of interventions, such as oral care, that may improve this dismal prognosis.

This cohort study provides new information to the sparse knowledge of the clinical significance of oral diseases in patients with cirrhosis that we had sought earlier.^42^ In conclusion, the patients had a high prevalence of various oral diseases, which was associated with the higher presence of cirrhosis complication status and higher CRP and nutritional risk score. Moreover, during follow‐up, the presence of more than one oral disease was associated with higher all‐cause and cirrhosis‐related mortality, markedly increasing with the number of oral diseases present. These findings highlight the need for mechanistic research and intervention studies that will probably lead to improved clinical course and prognosis of cirrhosis.
